# Retraction: Genetically Engineered Synthetic Miniaturized Versions of *Plasmodium falciparum* UvrD Helicase Are Catalytically Active

**DOI:** 10.1371/journal.pone.0158859

**Published:** 2016-06-30

**Authors:** The PLOS ONE Editors

The PLOS ONE editors retract this article following an investigation into concerns about the presentation of Figures 6 and 10.

The PLOS ONE editors were alerted to concerns about Fig 6 and 10 in the article. Upon follow up with the authors and an evaluation of the raw data supplied, concerns about the figures remained and an institutional investigation was requested.

The investigation conducted by the International Centre for Genetic Engineering and Biotechnology revealed that figures 6 and 10 show evidence of inappropriate manipulation: Fig 6 panels C and D are derived from the same gel; Fig 10 A, B and C are derived from the same gel. Additionally, the substrate structure as described in the text is missing from Fig 10.

Consistent with the recommendation by the International Centre for Genetic Engineering and Biotechnology of the investigation panel, the PLOS ONE editors retract this publication.
